# Complications associated with subpalpebral lavage systems in upper and lower equine eyelids: A prospective, randomised study in 73 cases (2015–2024)

**DOI:** 10.1111/evj.14540

**Published:** 2025-06-26

**Authors:** Annabelle E. Graham, Harry B. Carslake, Fernando Malalana

**Affiliations:** ^1^ Institute of Infection, Veterinary and Ecological Sciences University of Liverpool Liverpool UK

**Keywords:** complications, eyelid, horse, SPL, subpalpebral lavage

## Abstract

**Background:**

Evidence for optimal location of subpalpebral lavage (SPL) systems is lacking.

**Objectives:**

To compare the rate and types of complications with SPL systems located in central upper‐ compared with medial lower‐eyelid in hospitalised patients.

**Study Design:**

Prospective, randomised treatment trial.

**Methods:**

Horses admitted for ophthalmic treatment using an SPL system from February 2015 to January 2024 were included if ocular pathology did not necessitate SPL system placement in a specific location. Coin toss was used to determine location. SPL systems were monitored at least daily, and complications were defined as major (displacement of footplate from the fornix ± corneal ulceration; loss of footplate; eyelid infection/abscess formation) or minor (loss of suture/tape; palpebral swelling; leakage or tube rupture; loss of injection port; subcutaneous swelling/abscess at suture site). Data were analysed using logistic regression.

**Results:**

Seventy‐three SPL systems in 68 horses were included, with 38 (52%) located in the upper and 35 (48%) in the lower eyelid, for a median (IQR) duration of 13 (8–16) days. Sixty‐nine complications (37 (54%) in upper and 32 (46%) in lower SPL systems) occurred in 44/73 (60%) of all SPL systems. Major complications occurred with 2 lower (2/69; 3%) and 10 upper SPL systems (10/69;14%). The most common major complication was displacement of the lavage footplate from the conjunctival fornix (7/69; 10%). The most common minor complication was loss of suture or butterfly tape (21/69; 30%). Treatment with chloramphenicol (OR = 0.3; 95% CI: 0.09–0.8; *p* = 0.02) or cross‐linked modified hyaluronic acid (OR = 3.9; 95% CI: 1.2–13.3; *p* = 0.03) was associated with any complication on multivariable analysis. Multivariable analysis showed that upper systems were 5.1 (95% CI: 1.0–25.7; *p* = 0.05) more likely to have a major complication than lower SPL systems.

**Main Limitations:**

Small study size.

**Conclusions:**

SPL system location had no effect on all complications, but major complications were more common in those placed in the upper lid.

## 1 INTRODUCTION

The use of subpalpebral lavage (SPL) systems is widely reported in horses as a method of topical treatment for ophthalmic conditions.^1^, ^2^, ^3^, ^4^, ^5^ They are particularly useful in cases with ocular pain, risk of globe rupture, or poor patient compliance.^3^, ^6^ The conjunctival fornix of either the upper or lower eyelid can be used for placement of SPL systems.^3^ The treatment tube then runs across the head and then caudally along the neck, to allow for medication administration distally at an injection port in the cervical region.^3^ Serious complications (corneal ulceration and palpebral cellulitis or infection) have been described following SPL system placement.^1^, ^5^ Commercially available kits have fewer reported complications in horses compared with previously described practice‐made lavage systems.^2^, ^4^, ^5^, ^7^ However, there are only two published retrospective case series that discuss the use and complication rate of commercially available SPL systems.^4^, ^5^ Despite their widespread use, the strength of evidence supporting the preferred location of the SPL system footplate is weak.^8^ Current recommendations are based on clinician preference, location of the ocular lesion, and degree of eyelid inflammation.^3^, ^4^ Two retrospective studies comparing the rate of complications associated with SPL systems in the upper and lower eyelids have been published as abstracts, but without details of the complications.^7^, ^9^ Quere and Chahory published the first study comparing upper and lower lid SPL system placements in a retrospective study design. They found a greater incidence of complications in the upper lid; however, the results were not statistically significant.^4^ Being a retrospective study, limitations included an absence of randomisation of SPL system placement, incomplete records, and unrecorded operator (intern, resident, or boarded specialist) for SPL system placement.^4^ The aim of this prospective, randomised treatment trial was to describe the complications associated with SPL systems located in either the upper or lower eyelid in hospitalised patients in a university hospital referral setting. Our hypothesis was that there would be no difference in the incidence of SPL system complications in the upper compared with lower eyelid.

## 2 MATERIALS AND METHODS

A convenience sample was used for the study, the owners of all horses admitted to Philip Leverhulme Equine Hospital for ophthalmic treatment using an SPL system from February 2015 to January 2024 were invited to participate, therefore an a priori sample size calculation was not performed. All horses had a full ophthalmic examination by a boarded internal medicine specialist before SPL system placement. Cases were excluded if, in the opinion of the attending clinician, the ocular pathology necessitated placement of the SPL in a specific location. A coin toss was used to randomly determine the SPL system location (upper central or lower medial lid). A commercially available 5F (MILA International Inc.) eye lavage kit with 150 cm silicone tubing and footplate was used for all cases. All SPL systems were placed routinely as described by Dwyer by either a boarded specialist, resident, or intern under direct supervision of a boarded specialist.^3^ The periorbital region and areas for sutures on the face were clipped and scrubbed with a 1:50 povidine/iodine:saline solution. Topical tetracaine was applied to the corneal surface before SPL system placement in all cases. Mepivacaine was used in all cases to perform a palpebral nerve block and subcutaneously for all butterfly stent sites and the exit of the tubing from either the central upper or medial lower eye lid. Zinc oxide tape was used for all ‘butterfly’ wings to attach the tubing to the face with a minimum of two tape butterfly stents per SPL system. Data relating to eyelid swelling, ocular comfort, suture security, patency, and proper location were collected at SPL placement and during hospitalisation. SPL systems were assessed at every drug administration, and by a veterinary surgeon at least once daily. Data collected included age, breed, sex, reason for SPL system, duration of clinical signs before SPL system placement, duration of SPL system in place, whether the horse was discharged with the SPL system in place, operator placing the SPL system, left or right eye, location of SPL system (upper or lower lid), whether an eye mask (Equivet eye protector) was fitted, if an infusion device was used, ophthalmic medication administered via the SPL system and type of complication(s). No follow‐up data was recorded for horses that went home with the SPL system in place. If replacement of an SPL system was required due to a complication, the second SPL system was not included in the study.

## 3 DATA ANALYSIS

Data analysis was performed using Excel version 16.77.1 (Microsoft) and SPSS version 24 (IBM). Complications recorded were categorised as either major (displacement of the footplate from the fornix with or without corneal ulceration; loss of footplate; eyelid infection/abscess formation) or minor (loss of suture or tape; palpebral cellulitis; leakage or tube rupture; loss of injection port; subcutaneous swelling or abscess at suture site). Continuous data were assessed for distribution using the Shapiro–Wilk test and presented as mean ± standard deviation. If the data was not normally distributed, median ± interquartile ranges were used. Univariable logistic regression was performed to identify risk factors associated with any complication and major complication as binary outcomes. Variables with *p* ≤ 0.25 were included in multivariable logistic regression models which were constructed using a backward stepwise elimination procedure. Before multivariable analysis, all variables were assessed for collinearity using Spearman's rank correlation coefficient (*r*_*s*_). For pairs with *r*_*s*_ > 0.8, the most statistically significant or biologically plausible variables from the univariable model were selected for the multivariable model. Univariable and multivariable logistic regression was also performed as described above for each complication category. Goodness‐of‐fit of multivariable logistic regression models were determined using the Hosmer–Lemeshow test and/or ROC curves.

## 4 RESULTS

**FIGURE 1:**
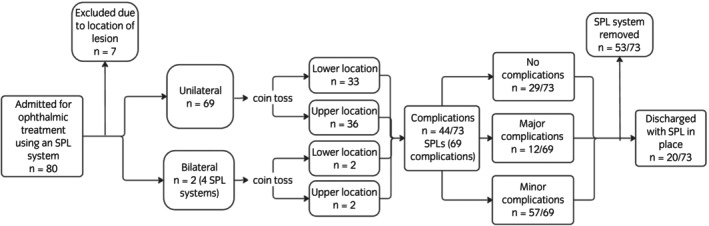
Flow diagram showing exclusion of cases, distribution of upper and lower SPL system locations and outcomes. No horses were excluded due to lack of consent.

There were 73 SPL systems in 71 eyes in 68 horses included in the study (Figure 1). There were 23 mares, 43 geldings, and 2 intact males. Mean (±SD) age was 12.1 ± 5.1 years (range 7 months to 24 years). Breeds were Cob/cob cross (*n* = 15), Thoroughbred/Thoroughbred cross (*n* = 13), Welsh (section A–D) (*n* = 11), Warmblood/Warmblood cross (*n* = 7), Irish Sports Horse (*n* = 6), Connemara (*n* = 4), Arabian (*n* = 2), Irish draught/Irish Draught cross (*n* = 3), Haflinger (*n* = 2), Shetland (*n* = 2), Dales (n = 1), Polo (*n* = 1), and unknown (*n* = 1).

Two horses had bilateral SPL systems placed, which were included as independent systems in the analysis. Three horses had two SPL systems placed at different times in the same eye, with a minimum duration of 99 days (mean (±SD) 250 (±134) days) between removal of the first SPL system and placement of the next, and these were also treated as independent SPL systems.

Thirty‐three (45%) SPL systems were placed in the right eye and 40 (55%) in the left eye. The SPL system was in the upper lid in 38/73 (52%) and the lower lid in 35 (48%) of cases (Table 1). A mask was fitted for 64 (88%) SPL systems. No SPL systems used an infusion pump. Subpalpebral lavage systems were in place for a median of 10.0 days (IQR 8–16) (range 3–58 days). The SPL system was removed before discharge in 53/73 (73%) of cases. The most frequent indication for SPL placement was bacterial corneal ulcer (31/73; 42%) and SPL systems were most frequently placed by residents (49/73; 67%).

**TABLE 1:** Location, duration of placement, complications, and operator for each indication for SPL placement.

Primary indication	Frequency	Location		Complications (any)		Complications (major)		Operator			Median duration (days)
		Upper	Lower	Upper	Lower	Upper	Lower	Intern	Resident	Clinician	
Bacterial corneal ulcer	31	15	16	18	18	4	1	7	21	3	12
Fungal corneal ulcer	6	5	1	4	0	0	0	0	6	0	10
Primary uveitis	12	6	6	8	6	4	0	6	6	0	8.5
Stromal abscess	4	3	1	2	1	1	0	1	3	0	14
Squamous cell carcinoma	9	3	6	1	4	0	1	1	5	3	10
Intraocular surgery	8	3	5	0	3	0	0	0	6	2	9
Viral corneal ulcer	3	3	0	4	0	1	0	1	2	0	22
Total number SPL systems	73	38	35	37	32	10	2	16	49	8	—

Horses received an average of 2.7 (±1) medications (±SD) through the SPL system. The most commonly used medication was chloramphenicol (0.5%; Martindale Pharma) (46/73; 63%), followed by atropine (1% atropine sulphate minims; Bausch&Lomb) (31/73; 43%) and EDTA (30/73; 41%), ciprofloxacin (0.3%, Ciloxan; Alcon) (21/73; 29%) and cross‐linked modified hyaluronic acid (Remend; Dômes Pharma) (21/73; 29%).

No complications were recorded in 29 SPL systems (29/73; 40%). Sixty‐nine complications occurred in 44 (60%) SPL systems, with 37/69 complications (54%) occurring in SPL systems at the upper eyelid location and 32/69 (46%) occurring in SPL systems in the lower eyelid. The number and type of complications are shown in Table 2. More than one complication occurred in 16/73 (22%) SPL systems.

**TABLE 2:** Location, operator, eye, and duration of placement for each category of complication following SPL placement.

Complication	Frequency of complication in all SPL systems	Location		Operator			Eye		Median duration (days)
		Upper	Lower	Intern	Resident	Clinician	Left	Right	
Major									
Displacement of footplate from the fornix	7	6	1	2	5	0	2	5	10
Loss of footplate	0	0	0	0	0	0	0	0	—
Eyelid infection/abscess formation	5	4	1	1	3	1	3	2	10
Minor									
Eyelid swelling	15	10	5	2	12	1	10	5	14
Subcutaneous swelling/abscess	2	0	2	0	1	1	1	1	43
Loss of suture	21	6	15	3	15	3	10	11	13
Loss of port	9	5	4	4	4	1	5	4	8
Leakage/tube rupture	10	6	4	4	3	3	5	5	11.5
Total complications	69	37	32	16	43	10	36	33	

Multivariable logistic regression models are detailed in Table 3. Any complication showed a significant association with chloramphenicol (OR 0.3; 95% CI: 0.09–0.8; *p* = 0.02) and treatment with cross‐linked modified hyaluronic acid (OR 5.4; 95% CI: 1.5–19.4; *p* = 0.01). Multivariable logistic regression analysis for major complications identified a significant association with upper location of the SPL system (OR 5.1; 95% CI: 1.0–25.7; *p* = 0.05). Univariable and multivariable logistic regression were performed for each type of complication. The variables with *p* < 0.25 are listed in Table S1 and *p* < 0.05 in Table 3 for univariable and multivariable logistic regression, respectively.

**TABLE 3:** Significant variables on multivariable logistic regression models for complications in 73 SPL systems.

Complication	Variable	Category	Frequency (%) with no complication	Frequency (%) with complication	Odds ratio	95% CI	*p*‐value	Goodness of fit	
								Area under ROC curve	Hosmer Lemeshow test *p*‐value
Any complication (*n* = 69)	Use of chloramphenicol via SPL	Yes	22 (48%)	24 (52%)	0.3	0.09–0.8		0.7	0.5
		No	7 (26%)	20 (74%)	Ref		0.02		
	Use of cross‐linked modified hyaluronic acid via SPL	Yes	4 (19%)	17 (81%)	5.4	1.5–19.4			
		No	25 (48%)	27 (52%)	Ref		0.01		
Major complication (*n* = 12)	Location	Upper	29 (76%)	9 (24%)	5.1	1.0–25.7		0.7	—
		Lower	33 (94%)	2 (6%)	Ref		0.05		
Eyelid swelling (*n* = 15)	Use of cross‐linked modified hyaluronic acid via SPL	Yes	13 (62%)	8 (38%)	4.0	1.2–13.0		0.7	—
		No	45 (87%)	7 (13%)	Ref		0.02		
Loss of suture (*n* = 21)	Age of horse	Years			1.2	1.1–1.4	0.005	0.8	0.6
	Location	Upper	32 (84%)	6 (16%)	0.2	0.06–0.7			
		Lower	20 (57%)	15 (43%)	Ref		0.01		
Leakage/tube rupture (*n* = 10)	Use of topical prednisolone via SPL	Yes	7 (64%)	4 (36%)	7.7	1.3–44.3		0.8	0.7
		No	56 (90%)	6 (10%)	Ref		0.02		
	Operator placing SPL	Clinician	5 (62%)	3 (38%)	15.3	2.0–114.0			
		Intern	12 (75%)	4 (16%)	3.7	0.6–21.1			
		Resident	46 (94%)	3 (6%)	Ref		0.03		

## 5 DISCUSSION

This is the first prospective, randomised study evaluating complications associated with SPL system footplate location. Overall complication frequency was similar in the upper‐ compared with lower‐lid location, although a difference was demonstrated for the individual complication of loss of suture, which was more frequent in the lower location. Complications categorised as major occurred more commonly with SPL systems located in the upper eyelid location.

Comparison of complication rates between studies can be challenging due to differences in the categorisation of complications. The categorisation of complications into minor and major groups was performed in part to allow for comparison to three previous studies.^1^, ^4^, ^6^ Major complications in other studies were defined as complications which necessitate SPL system removal (including iatrogenic corneal ulceration, palpebral abscess leading to a chemosis and purulent secretions at the end of the catheter in the eyelid, and the overgrowth of conjunctiva over the footplate).^1^, ^4^, ^6^ Minor complications were defined as palpebral cellulitis, tearing of the SPL system tubing, and loss of the injection cap. Two other studies defined complications as ocular and non‐ocular.^2^, ^5^ This categorisation has significant overlap as most ocular and non‐ocular complications would have been categorised as major and minor, respectively. However, in these two studies, moderate to severe swelling and cellulitis of the eyelid was considered an ocular complication.^2^, ^5^ Therefore, comparison of their results to this article requires caution.

The overall complication rate in this study was higher than previously reported for other studies using commercial SPL systems. However, given the prospective nature of the study, it is more likely that less clinically important complications, such as loss of an injection port or sutures, were reported. However, the major complication rate was similar to other studies. In the upper location, the major complication rate (10/37; 27%) was comparable to the reported range of 21.2%–27%.^1^, ^4^, ^5^ In the lower location, the major complication rate (2/32; 6%) was also comparable to the reported range 3%–4.2%.^2^, ^4^

Evidence for the differences in the complications of SPL systems in the upper‐ versus lower‐eyelid location in the existing literature is limited and conflicting.^2^, ^4^, ^8^ More upper location complications were found by Quéré and Chahory, but this difference was not significant.^4^ This current study is the first to show that the upper location is significantly more likely to have a major complication compared with the lower location. This difference has previously been hypothesised to be due to anatomical differences that result in displacement of the footplate.^2^ In the current study, 6/7 displaced footplates were in the upper location, but location was not a significant risk factor for this complication in the multivariable analysis. This may be because the study lacked statistical power. Older horses were found to have an increased risk of suture loss in the multivariable analysis. However, the authors cannot identify a biologically plausible explanation for this finding. This study identified a reduced risk of suture loss with the SPL system in the upper eyelid location. This is likely because fewer butterfly stents and therefore sutures are required for placement in the upper location. Subcutaneous tunnelling of the SPL tubing (for 1–2 cm at two or three locations across the face) has also been anecdotally described by some clinicians to avoid this complication. However, risks of complications associated with this method have not been evaluated.

There are two studies that have evaluated complications associated with antimicrobials and serum administered through SPL systems, but their evidence is conflicting.^5^, ^7^ Cornelissen et al. presented an abstract that showed complications were reported in 13.2% of SPLs when topical antimicrobial drugs were used, compared with 25% of SPLs without antimicrobials (*p* = 0.008).^7^ No difference was found by Stewart.^5^ In this study, antimicrobial drugs were used in 62/73 (85%) SPL systems, with a reduced odd ratio of any complication if chloramphenicol was used. However, given the very low number of SPLs in which no antimicrobial was administered, this should be interpreted with caution. The use of ciprofloxacin was predominantly reserved for corneal ulceration or stromal abscesses (16/21; 76%). As a referral hospital, cases of corneal ulceration are usually referred for poor response to initial treatment, usually with a first‐line antimicrobial such as chloramphenicol. In most of the corneal ulceration cases (bacterial, fungal, or viral) chloramphenicol was still used, whilst ciprofloxacin was reserved for the more refractory cases, or when guided by culture and susceptibility testing. There were five SPL systems where both antimicrobials were used at separate times during treatment. Data regarding bacterial culture and susceptibility was not recorded to confirm if this was based on susceptibility results. However, the use of ciprofloxacin can be justified in select, complex referral cases.^10^ Cornelissen et al. also found that plasma given via a SPL system was associated with an increased complication rate (*p* = 0.004; 30.4% when used; 13.0% when not).^7^ Stewart et al. identified a correlation between increased complications in equine SPL systems and the use of equine serum.^5^ However, equine serum was only used in two cases in this current study so this relationship could not be examined. Equine serum was infrequently used as it is the authors' preference to use EDTA over serum as it has been shown to have more effective inhibitory effects on matrix metalloproteinases 2 and 9 than equine serum.^11^, ^12^

The use of cross‐linked modified hyaluronic acid (HA) was associated with the development of any complication and eyelid swelling in the multivariable analyses. The use of HA as a topical tear replacement has been widely reported in veterinary literature.^13^ The use of cross‐linked HA has also been shown to decrease corneal healing times in small animals; therefore, HA was used predominantly in this current study as an adjunct treatment for corneal ulceration.^14^ However, to the authors' knowledge, the use of HA in SPL systems has not been evaluated. Formulations of HA demonstrate thixotropic properties, with lower viscosity under high shear forces, such as blinking, but higher viscosity under low shear forces.^15^ Crosslinking HA enhances the viscoelastic properties to extend the contact time of the HA with ocular surfaces.^13^ However, the effects of HA viscoelastic properties within SPL tubing system have not been evaluated to the authors' knowledge. Low velocity of HA within the tubing could hypothetically lead to accumulation within the tube, but it is unclear how that would relate to the other complications reported in this study. Whether medication was stacked (SPL system tubing pre‐loaded with medication) within an SPL system tube or air‐flushed following each drug was not recorded in this study. Although both techniques are used on a case‐dependent basis, depending on the horse's tolerance of air‐flushing medication, further research is needed to determine the effects of HA within SPL tubing and the significance for SPL system complications.

The major limitation of this study was the small sample size. Fewer cases were recruited than anticipated, and the overall complication rate was greater than expected across both groups, meaning that a larger sample size would be required to detect a significant difference. A convenience sample was used as previous study data was not equivalent, as they used non‐commercial SPL systems and were only positioned in one location.^1^, ^2^ Therefore, although no statistically significant difference was detected between SPL footplate locations for any individual complication, the results cannot conclude that it is because no difference exists. The only other study that has evaluated upper compared with lower complication rates was unpowered to show a statistically significant difference.^4^ Whilst this current study had a greater degree of power for major complications than Quéré and Chahory,^4^ due to the prospective design and increased incidence of minor complications, a multicentre study would be needed to increase case numbers. However, the prospective design adds further higher quality evidence to other studies for the overall complication rates for upper and lower lid. A further limitation was that cytology, bacteriology, and viral PCR data was not recorded. Therefore, it was not possible to determine if cases recorded as bacterial, viral, or fungal corneal ulcers were based on positive culture, cytology, PCR, or clinical suspicion of a specific aetiology. This study also only examined complications associated with SPL systems and not efficacy of drug distribution. A study in people showed that fluorescein applied under the upper eyelid had greater contact time and increased duration of absorption compared with application under the lower lid.^16^ Application of topical medication increases tear volume and stimulates blinking, which is presumed to help distribute the drug across the corneal surface when applied under the lower lid.^17^ However, blinking can also contribute to increased medication losses by forcing tears into the puncta in the corners of the upper and lower eyelids, resulting in increased drainage.^17^, ^18^ However, the effects of blinking and location of ocular medication on drug distribution and absorption have not been evaluated in horses.

## 6 CONCLUSION

This is the first prospective, randomised study evaluating complications associated with SPL system location. Overall complication frequency was similar in the upper‐ compared with lower‐lid systems, although a difference was demonstrated for the individual complication of loss of suture. Complications categorised as major occurred more commonly with SPL systems located in the upper lid and may be more likely to require early removal of the SPL system. Therefore, a lower lid footplate location could be preferential if close monitoring of the SPL system is not possible, such as if the horse was discharged with the SPL system in place. Larger, multicentre studies are required to further examine risk for specific complications. These data will help guide clinical decision‐making around the placement of SPL systems.

## FUNDING INFORMATION

Nothing to declare.

## CONFLICT OF INTEREST STATEMENT

The authors have declared no conflicting interests.

## AUTHOR CONTRIBUTIONS

**Annabelle E. Graham:** Writing – original draft; investigation; methodology; writing – review and editing; software; formal analysis; project administration; data curation; visualization. **Harry B. Carslake:** Conceptualization; investigation; writing – review and editing; methodology; supervision; visualization; resources. **Fernando Malalana:** Conceptualization; investigation; methodology; writing – review and editing; formal analysis; supervision; visualization; resources.

## DATA INTEGRITY STATEMENT

Annabelle E. Graham and Fernando Malalana had full access to all the data in the study and take responsibility for the integrity of the data and the accuracy of the data analysis.

## ETHICAL ANIMAL RESEARCH

The study was approved by the University of Liverpool's committee on research (ethical approval VREC292).

## INFORMED CONSENT

Owners gave consent for their animals' inclusion in the study.

## Supporting information


**Table S1.** Significant variables on univariable logistic regression models for any complication, major complication, or individual type of complication in 73 SPL systems. All drugs stated as variables were used topically via the subpalpebral lavage systems.

## Data Availability

The data that support the findings of this study are openly available in University of Liverpool Datacat at https://datacat.liverpool.ac.uk/2873, reference number 2873.
