# Supplementary material for: Complications associated with subpalpebral lavage systems in upper and lower equine eyelids: A prospective, randomised study in 73 cases (2015–2024)
Source: Equine Vet J. 2025 Jun 26;58(3):692–8. doi: 10.1111/evj.14540 (PMC13041592; doi:10.1111/evj.14540)
Supplement: Supplementary file 1 — Table S1. Significant variables on univariable logistic regression models for any complication, major complication, or individual type of complication in 73 SPL systems. All drugs stated as variables were used topically via the subpalpebral lavage systems. [file EVJ-58-692-s001.pdf]

Equine Veterinary Journal BEVA

Table S1: Significant variables on univariable logistic regression models for any complication, major complication or individual type of complication in 73 SPL systems. All drugs stated as variables were used topically via the subpalpebral lavage systems.

Complication	Variable	Category	Total number	Complication occurred		Odds ratio	95%CI		*p*-value
				No	Yes				
**Any complication (n=69 affecting 44 SPLs)**	Indication	Bacterial corneal ulcer	31	12	19	REF			0.2
		Fungal corneal ulcer	6	4	2	0.3	0.05	2.0	
		Primary uveitis	12	1	11	7.0	0.8	60.9	
		Stromal abscess	4	1	3	1.9	0.2	20.4	
		Squamous cell carcinoma	4	5	4	0.5	0.1	2.3	
		Intraocular surgery	8	5	3	0.4	0.08	1.9	
		Viral corneal ulcer	3	1	2	1.3	0.10	15.5	
	Use of chloramphenicol	Yes	46	22	24	0.4	0.1	1.1	0.07
		No	27	7	20	REF			
	Use of prednisolone	Yes	11	1	10	8.2	1.0	68.3	0.05
		No	62	28	34	REF			
	Use of cross-linked modified hyaluronic acid	Yes	21	4	17	3.9	1.2	13.3	0.03
		No	52	25	27	REF			
	Use of voriconazole	Yes	11	7	4	0.3	0.08	1.1	0.09
		No	62	22	40	REF			
	Use of dorzolamide/timolol	Yes	4	3	1	0.2	0.02	2.0	0.2
		No	69	26	43	REF			
**Major complication (n=12)**	Eye	Right	33	26	7	2.4	0.6	9.1	0.2
		Left	40	36	4	REF			
	Location	Lower	35	33	2	0.2	0.04	1.0	0.05
		Upper	38	29	9	REF			
	Use of prednisolone	Yes	11	8	3	2.5	0.6	11.6	0.23
		No	62	54	8	REF			
	Use of dexamethasone/ polymixin B/neomycin (combination product)	Yes	5	3	2	4.4	0.6	29.9	0.1
		No	68	59	9	REF			
	Use of atropine	Yes	31	24	7	2.8	0.7	10.5	0.1
		No	42	38	4	REF			
	Use of phenylephrine	Yes	6	4	2	3.2	0.5	20.2	0.21
		No	67	58	9	REF			
	Eye	Right	33	28	5	0.3	0.1	1.6	0.1

**Displacement of footplate from the fornix (n=7)**		Left	40	38	2	REF			
	Location	Lower	35	34	1	0.2	0.0	1.4	0.10
		Upper	38	32	6	REF			
	Mask fitted	Yes	64	59	5	0.3	0.0	1.8	0.2
		No	9	7	2	REF			
	Use of chloramphenicol	Yes	46	44	2	5.0	0.9	27.9	0.07
		No	27	22	7	REF			
	Use of prednisolone	Yes	11	8	3	5.4	1.0	28.9	0.05
		No	62	58	4	REF			
	Use of dexamethasone/ polymixin B/neomycin (combination product)	Yes	5	3	2	8.4	1.1	62.5	0.04
		No	68	63	5	REF			
	Use of phenylephrine	Yes	6	4	2	6.2	0.9	42.6	0.06
		No	67	62	5	REF			
**Eyelid swelling (n=12)**	Discharged in place	Yes	20	18	2	0.3	0.07	1.7	0.2
		No	53	40	13	REF			
	Location	Lower	35	30	5	0.5	0.1	1.5	0.21
		Upper	38	28	10	REF			
	Use of atropine	Yes	31	28	3	0.3	0.07	1.1	0.06
		No	42	30	12	REF			
	Use of cross-linked modified hyaluronic acid	Yes	21	13	8	4.0	1.2	13.0	0.02
		No	52	45	7	REF			
	Use of gancyclovir	Yes	3	1	2	8.8	0.7	104.2	0.09
		No	70	57	13	REF			
**Eyelid infection (n=12)**	Duration in place					1.1	1.0	1.1	0.09
	Use of gancyclovir	Yes	3	2	1	8.3	0.6	111.5	0.1
		No	70	66	4	REF			
**Subcutaneous swelling/ abscess at site of skin sutures (n=2)**	Age					1.3	1.0	1.8	0.1
	Duration in place					1.2	1.0	1.3	0.01
	Use of serum	Yes	3	2	1	34.5	1.5	771.9	0.03
		No	70	69	1	REF			
**Loss of suture(s) (n=21)**	Age					1.2	1.1	1.3	0.24
	Sex	Gelding	45	29	16	0.9	0.08	10.8	
		Stallion	3	2	1	0.4	0.1	1.2	
		Mare	25	21	4	REF			
	Duration in place					1.1	1.0	1.1	0.03
	Discharged in place	Yes	20	12	8	2.1	0.7	6.1	0.2
		No	53	40	13	REF			
	Location	Lower	35	20	15	4.0	1.3	12.0	0.01

		Upper	38	32	6	REF			
	Use of serum	Yes	3	1	2	5.4	0.5	62.7	0.2
		No	70	51	19	REF			
	Use of ethylenediamine tetraacetic acid (EDTA)	Yes	30	17	13	3.4	1.2	9.6	0.03
		No	43	35	8	REF			
**Loss of injection port (n=9)**	Operator	Clinician	8	7	1	1.6	0.2	16.5	0.2
		Intern	16	12	4	3.8	0.8	17.2	
		Resident	49	45	4	REF			
**Leakage/tube rupture (n=10)**	Operator	Clinician	8	5	3	9.2	1.5	58.4	0.04
		Intern	16	12	4	5.1	1.0	26.0	
		Resident	49	46	3	REF			
	Duration in place					1.1	1.0	1.1	0.09
	Use of prednisolone	Yes	11	7	4	5.3	1.2	23.7	0.03
		No	62	56	5	REF			
